# Sparse 3D contrast-enhanced whole-heart imaging for coronary artery evaluation

**DOI:** 10.1007/s00059-021-05091-6

**Published:** 2022-01-10

**Authors:** Uzair Ansari, Sonja Janssen, Stefan Baumann, Martin Borggrefe, Stephan Waldeck, Stefan Schönberg, Theano Papavassiliu, Daniel Overhoff

**Affiliations:** 1 First Department of Medicine, University Medical Center Mannheim, Faculty of Medicine Mannheim, University of Heidelberg, Theodor-Kutzer-Ufer 1–3, 68167 Mannheim, Germany; 2 European Center for AngioScience (ECAS), Mannheim, Germany; 3 DZHK (German Center for Cardiovascular Research) Partner Site Heidelberg/Mannheim, Mannheim, Germany; 4 Institute of Clinical Radiology and Nuclear Medicine, University Medical Center Mannheim, Medical Faculty Mannheim, Heidelberg University, Heidelberg, Germany; 5 Department of Radiology and Neuroradiology, Bundeswehr Central Hospital Koblenz, Koblenz, Germany

**Keywords:** Coronary artery disease, Whole-heart imaging, Noninvasive MR coronary angiography, Koronare Herzkrankheit, Ganzherzbildgebung, Nicht invasive MR-Koronarangiographie

## Abstract

**Background:**

We investigated the feasibility of evaluating coronary arteries with a contrast-enhanced (CE) self-navigated sparse isotropic 3D whole heart T1-weighted magnetic resonance imaging (MRI) study sequence.

**Methods:**

A total of 22 consecutive patients underwent coronary angiography and/or cardiac computed tomography (CT) including cardiac MRI. The image quality was evaluated on a 3-point Likert scale. Inter-reader variability for image quality was analyzed with Cohen’s kappa for the main coronary segments (left circumflex [LCX], left anterior descending [LAD], right coronary artery [RCA]) and the left main trunk (LMT).

**Results:**

Inter-reader agreement for image quality of the coronary tree ranged from substantial to perfect, with a Cohen’s kappa of 0.722 (RCA_mid_) to 1 (LCX_prox_). The LMT had the best image quality. Image quality of the proximal vessel segments differed significantly from the mid- and distal segments (RCA_prox_ vs. RCA_dist_, *p* < 0.05). The LCX segments showed no significant difference in image quality along the vessel length (LCX_prox_ vs. LCX_dist_, *p* = n.s.). The mean acquisition time for the study sequence was 553 s (±46 s).

**Conclusion:**

Coronary imaging with a sparse 3D whole-heart sequence is feasible in a reasonable amount of time producing good-quality imaging. Image quality was poorer in distal coronary segments and along the entire course of the LCX.

**Supplementary Information:**

The online version of this article (10.1007/s00059-021-05091-6) contains supplementary material, which is available to authorized users.

## Abstract (de)

**Hintergrund:**

Die Durchführbarkeit einer Beurteilung der Koronararterien mit einer kontrastmittelverstärkten self-navigated sparse isotropen T1-gewichteten 3D Whole Heart Magnetresonanztomographie-Bildgebungssequenz (CE-MRT) wurde ermittelt.

**Methoden:**

Bei 22 konsekutiven Patienten wurde eine Koronarangiographie und/oder eine kardiale Computertomographie (CT) einschließlich kardialer MRT durchgeführt. Die Bildqualität wurde auf einer 3‑Punkt-Likert-Skala beurteilt. Mit dem Kappa-Koeffizienten nach Cohen für die Hauptkoronarsegmente (Ramus circumflexus der linken Koronararterie [LCX], Ramus interventricularis anterior [LAD], rechte Koronararterie [RCA]) und den linken Hauptstamm [LMT]) wurde die Inter-Reader-Variabilität für die Bildqualität ausgewertet.

**Ergebnisse:**

Mit einem Kappa-Koeffizienten nach Cohen von 0,722 (RCA_mid_) bis 1 (LCX_prox_) lag die Inter-Reader-Übereinstimmung für die Bildqualität des Koronarbaums zwischen beträchtlich und perfekt. Der LMT wies die beste Bildqualität auf. Die Bildqualität der proximalen Gefäßsegmente unterschied sich signifikant von den mittleren und den distalen Segmenten (RCA_prox_ vs. RCA_dist_; *p* < 0,05). Bei den LCX-Segmenten ergaben sich keine wesentlichen Unterschiede in der Bildqualität über die Länge des Gefäßes (LCX_prox_ vs. LCX_dist_; *p* = n.s.). Die mittlere Aufnahmezeit für die Untersuchungssequenz betrug 553 s (±46 s).

**Schlussfolgerung:**

Die Darstellung der Koronararterien mittels einer sparse kontrastmittelverstärkter 3D Whole Heart Sequenz ist in einem annehmbaren Zeitrahmen machbar, dabei wird eine Bildgebung von guter Qualität erzeugt. Eine schlechtere Bildqualität zeigte sich jedoch in distalen Koronarsegmenten und entlang des gesamten Verlaufs des LCX.

The diagnostic algorithm for suspected coronary artery disease (CAD) is centered around the stratification of risk and assessment of pre-test probability with the help of multiple determinants such as age, family history, symptoms, and basic clinical testing. This is then followed by use of noninvasive diagnostic tools, which could potentially establish CAD in an at-risk patient. Invasive coronary angiography (ICA), once considered as an essential component of this diagnostic pathway, is now deemed to be a reasonable option if ischemia is evident or the risk of cardiac events is high [1].

The European Society of Cardiology (ESC) has recently suggested the use of noninvasive anatomical imaging using coronary CT angiography (cCTA) to improve the diagnostic yield in patients with low-to-intermediate risk. However, the extensive use of cCTA is limited by routine availability, the application of an iodinated contrast agent, as well as radiation exposure [2].

Magnetic resonance coronary angiography (CMRA) is a viable noninvasive alternative to cCTA/ICA, which could aid in the diagnosis of CAD with good accuracy in the large and proximal branches of the coronary arteries [3]. As CMRA does not suffer from beam-hardening or “blooming” artifacts caused by high-density calcification, the method could potentially outline the lumen of heavily calcified arteries of the coronary tree [4]. The introduction of three-dimensional (3D) steady-state free-precision (SSFP) whole-heart CMRA for coronary imaging enables complete visualization of the coronary arterial tree within a single axial 3D acquisition [5]. Although, the spatial and temporal resolution of these images is still inferior to cCTA, this has been mostly attributed to the lower signal-to-noise ratio (SNR), especially seen in the 1.5‑T MRI systems. A significant limitation of CMRA is the comparatively lengthy acquisition times. The recent use of 3.0‑T MRI systems and 32-element coils potentially overcomes this limitation by improving the SNR and using parallel imaging techniques [6]. Moreover, free breathing practices and novel methods using 3D affine transformations on acquired data, processed retrospectively so as to reduce respiratory motion artifacts, have shown great promise [7].

The aim of this study was to investigate a self-navigated sparse isotropic 3D whole-heart T1-weighted sequence with an acceleration factor of around 10, in a clinical setting, and to evaluate image quality of the coronary arteries in comparison with the reference standard ICA or cCTA.

## Materials and methods

### Study participants and design

**Fig. 1:**
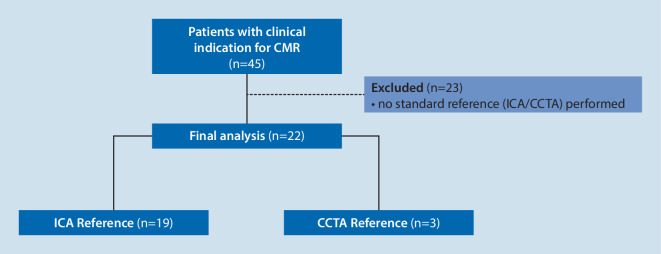
Algorithm for patient enrollment. *CMR* cardiovascular magnetic resonance imaging, *ICA* invasive coronary angiography, *ICA/CCTA* coronary computed tomography angiography

This single-center study was initiated with a group of 45 prospectively enrolled patients undergoing a clinically indicated contrast-enhanced (CE) cardiac MRI with the prescribed MR study sequence between March 2017 and November 2018. A total of 22 patients who further underwent an ICA and/or cCTA were included in the final analysis (Fig. 1).

The study included patients suspected of having CAD based on their medical history as well as those exhibiting signs and symptoms suggestive of obstructive coronary stenoses. Patients with general contraindications to CMRA (e.g., claustrophobia, metallic implants, allergy), as well as those with an acute myocardial infarction or history of coronary-artery bypass graft (CABG) surgery, were excluded from the study.

This study was approved by our local ethics committee (Medizinische Ethikkommmision II, Medizinische Fakultät Mannheim) and was conducted according to standards of the Health Insurance Portability and Accountability Act (HIPAA) and the Declaration of Helsinki.

### Data acquisition

The CMRA was performed on a 3‑T scanner system (Magnetom Skyra, Siemens Healthineers, Erlangen, Germany). Cardiac synchronization was achieved with vector electrocardiogram (ECG). Standard scout images were obtained in the axial, sagittal, and coronal views along with sequences performed in end-expiratory breath-hold covering the heart from base to apex according to retrospective ECG-triggered CMR protocols.

Cine images were acquired using a retrospective ECG-gated, balanced segmented SSFP (trueFISP) sequence in three long-axis views (two-, three-, and four-chamber view) and in multiple short-axis views, covering the entire left ventricle from base to apex. The study sequence itself featured a self-navigated sparse isotropic 3D whole-heart T1-weighted prototype sequence with an acceleration factor of around 10, acquired in the waiting time between contrast administration and acquisition of the late gadolinium enhancement (LGE) sequences.

**Fig. 2:**
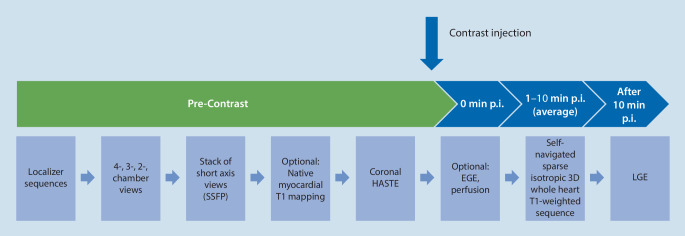
Sequence protocol overview for whole-heart CMR. *CMR* cardiovascular magnetic resonance imaging, *HASTE* half-Fourier single-shot turbo spin-echo sequence, *EGE* early gadolinium enhancement, *SSFP* steady-state free precession, *LGE* late gadolinium enhancement, *p.i.* post-injection

Body weight-adapted LGE images of the standard axis were acquired 10–15 min after i.v. injection of 0.2 mmol⋅kg^−1^ gadoteric acid (Dotarem, Guerbet, Roissy CdG Cedex, France, Germany). The myocardial signal was “nulled” by adjusting inversion recovery (IR) time individually for every patient, typically resulting in an IR time between 240 and 300 ms (Fig. 2).

### Whole-heart CMRA analysis

The CMRA analysis was conducted by two radiologists with more than 5 years of experience in reading cardiovascular magnetic resonance (CMR) and cCTA images. Both readers were blinded to the results of the reference standard ICA and cCTA, and independently evaluated the image quality of the coronary arterial tree based on a 3-point Likert scale (1 = *insufficient visualization*; 2 = *sufficient visualization*; 3 = *excellent visualization*). The right coronary artery (RCA) and the left anterior descending (LAD) artery were rated in the proximal, mid-, and distal segments. The left circumflex (LCX) was evaluated in the proximal and distal segments, and the left main trunk (LMT) as a whole.

**Fig. 3:**
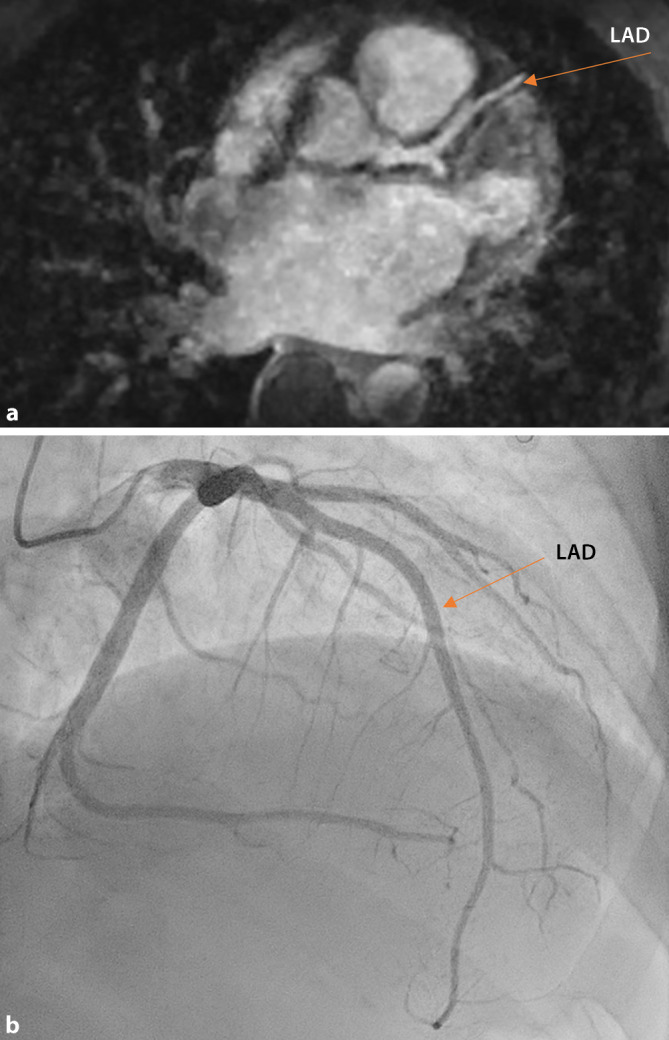
Coronary imaging using invasive coronary angiography and the whole-heart sequence in coronary magnetic resonance angiography (CMRA) for visualization of the coronary arterial tree. Self-navigated 3D CMRA. Whole-heart sequence (CMRA, **a**) with corresponding image from the invasive coronary angiography (coronary computed tomographic angiography, **b**) acquired from a patient revealing the proximal left anterior descending artery (LAD; see *arrow*)

**Fig. 4:**
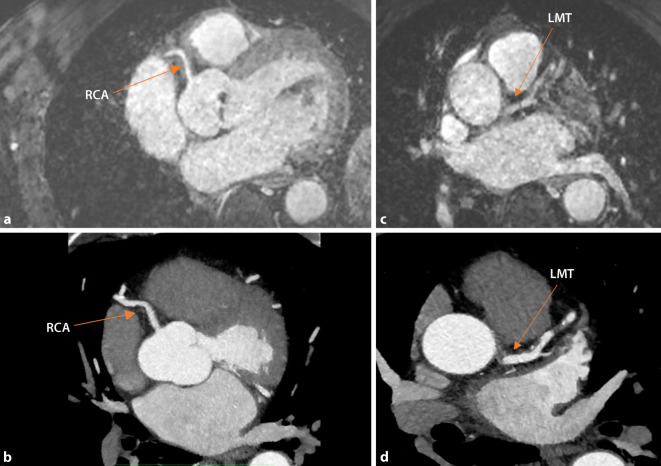
Coronary imaging using coronary CT angiography and coronary magnetic resonance angiography (CMRA). Whole-heart sequence for visualization of the coronary arterial tree. **a**, **b** Self-navigated 3D CMRA. Whole-heart sequence (CMRA, **a**) with corresponding coronary computed tomographic angiography (cCTA, **b**) acquired from a patient revealing the proximal right coronary artery (RCA). **c**, **d** Self-navigated 3D CMRA. Whole-heart sequence (CMRA, **c**) with corresponding cCTA (**d**) acquired from a patient revealing the left main trunk (LMT)

In the second step, both readers once again independently rated the image quality of the coronary arterial tree after 3 months, while comparing it with corresponding ICA/cCTA images. Images from patients are shown in Figs. 3 and 4.

### Conventional coronary angiography

Invasive coronary angiography was performed on the patients using the Judkins approach either through the radial or femoral route. All relevant projections acquired during the angiogram were evaluated by an experienced cardiologist who was blinded to the results of the CMRA. A significant coronary artery stenoses was defined as luminal narrowing in diameter greater than 50%.

### Coronary CT angiography

A third-generation dual-source CT (Siemens SOMATOM FORCE, Siemens Healthineers, Forchheim, Germany) was used for imaging. A specified regimen of medication consisting of sublingual nitroglycerin (0.8 mg) and intravenous beta-blockers were given prior to the scan if deemed necessary by a radiologist. An initial dose of 80 mL iodinated contrast material (Iomeron 400; Bracco Imaging S.p.A., Milan, Italy) was administered using a power injector. Coronary calcification was quantified using the Agatston score, which was calculated using a software application based on the Agatston scoring convention (syngo.via, syngo.CT CaScoring, Siemens Healthineers). The cCTA datasets were used for morphological plaque analysis and calculated by on-site prototype software (syngo.via Frontier, Coronary Plaque Analysis 2.0, Siemens Healthineers).

### Statistical analysis

Statistical analysis was performed using the Statistical Package for the Social Sciences (IBM SPSS Statistics, version 20.0 for Macintosh; SPSS, Inc., Chicago, IL, USA).

All continuous data are expressed as mean ± SD. The inclusion of variables for analysis was based on association (*p* < 0.05) in the Student *t-*test or clinical relevance. The differences between the image quality between various coronary segments were initially analyzed using the Mann–Whitney *U*-test. In the second step, the image quality was rated while comparing them with standard imaging from cCTA or ICA. Kappa coefficients were calculated to assess the degree of agreement for binary factors. Inter-reader variability for image quality was analyzed with Cohen’s kappa individually for every coronary segment of the three main branches (LCX, LAD, and RCA) as well as the LMT. The Wilcoxon test was used to compare the different methods and applied for results of significance on an ordinal scale. Values of *p* ≤ 0.05 were considered significant.

## Results

A total of 22 patients underwent both whole-heart sequences during the CMR as well as an ICA or cCTA. These were included in the study for final analysis after fulfillment of the eligibility criteria (Fig. 1). Free-breathing whole-heart CMRA acquisitions and reconstructions were successfully completed for all participants. The patient collective included 12 males and 10 females. The distribution of CAD in the patient population was evenly spread, with eight patients having normal coronary arteries, six patients having a triple-vessel disease, five patients with single-vessel disease, and three patients with coronary atherosclerosis without significant coronary stenoses. The median age of the patient population was 64 years (24–78 years; Supplementary table).

### Image quality of CMRA

The mean acquisition time for the study sequence was 553 ± 46 s. The CE whole-heart sequences of different patients are shown in Figs. 3 and 4.

**Fig. 5:**
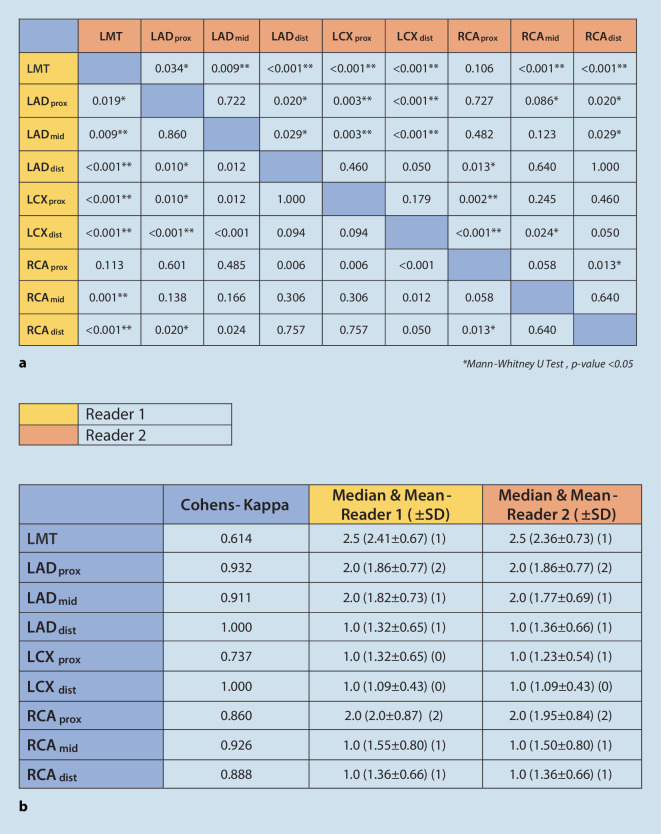
**a** Variation in quality of imaging across vessel length: first reading. **b** Quality of vessel imaging according to Likert scale: first reading. **a** Mann–Whitney *U*‑test, *p* < 0.05. *LMT* left main trunk, *LAD* left anterior descending, *LCX* left circumflex, *RCA* right coronary artery

The CMRA sets showed good quality imaging in all patients. Each reader during the first sitting showed a tendency to rate a better image quality for the proximal vessel segments compared to the mid- and distal segments. The LMT had superior images as a whole (2.41 ± 0.67/2.36 ± 0.73) with a significant difference in quality compared to distal ends of the coronary tree (e.g., LMT vs. LAD_dist_, *p* < 0.001 or LMT vs. RCA_dist_, *p* < 0.001). The readers rated the proximal LAD segments to have better image quality than the distal segments (Reader 1 LAD_prox_ vs. LAD_dist_, *p* < 0.01; Reader 2 LAD_prox_ vs. LAD_dist_, *p* < 0.02). Similar results were also seen for the proximal RCA segments (*p* = 0.013 for both readers). Interestingly, the LCX segments showed no significant difference in image quality along the course of the vessel length (LCX_prox_ vs. LCX_dist_, *p* = n.s. for both readers). This could also be attributed to the poor imaging of the entire vessel as suggested by mean values derived from the ratings of both readers (LCX_prox_ 1.32 ± 0.65/1.23 ± 0.54; LCX_dist_ 1.09 ± 0.43 for both). The inter-reader variability in the first sitting varied between a Cohen’s kappa index of 0.614 (for LM) and 1.0 (LAD_dist_ and LCX_dist_; (Fig. 5a, b).

**Fig. 6:**
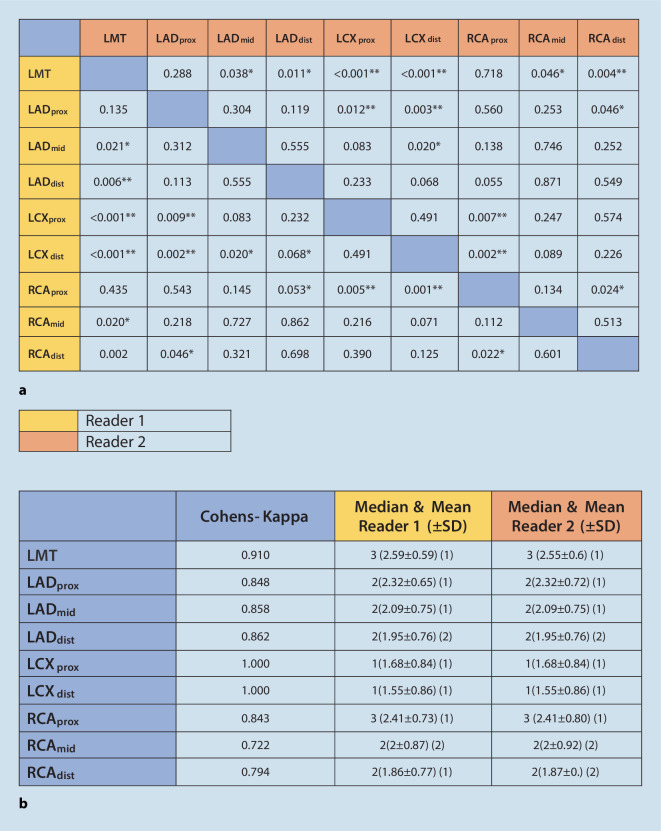
**a** Variation of quality of imaging across vessel length: second reading. **b** Quality of vessel imaging according to Likert scale: second reading *Asterisk* Mann–Whitney *U*-test, *p* < 0.05. *LMT* left main trunk, *LAD* left anterior descending, *LCX* left circumflex, *RCA* right coronary artery

In the second step, images rendered by the CMRA were compared with the standard reference images acquired in the cCTA and/or the ICA. The proximal branches of the LAD and the RCA showed better image quality, and this was not significantly different from the LMT (LMT vs. LAD_prox_ *p* = n.s.; LMT vs. RCA_prox_ *p* = n.s. for both readers). Imaging reproduced for the LAD showed good quality throughout the vessel length (*p* = n.s. for both readers). Nevertheless, the quality of coronary imaging did deteriorate significantly when proximal segments of the RCA were compared with the distal segments (Reader 1: RCA_prox_ vs. RCA_dist,_ *p* = 0.022; Reader 2: RCA_prox_ vs. RCA_dist,_ *p* = 0.024). The LCX continued to show no significant difference in image quality along the course of the vessel length (LCX_prox_ vs. LCX_dist_ *p* > 0.05). This could once again be attributed to the poor image quality of the vessel as a whole as suggested by the mean values (LCX_prox_ 1.68 ± 0.84; LCX_dist_ 1.55 ± 0.86 for both readers). The inter-reader variability in the second sitting varied between a Cohen’s kappa index of 0.722 (for RCA_mid_) and 1.0 (LCX_prox_ and LCX_dist_). A comparison of all segments revealed the best image quality for LMT with a mean value of 2.59 ± 0.81, whereas the distal LCX with a mean value of 1.55 ± 0.86 registered a poorer overall image quality rating (Fig. 6a, b).

The intra-reader variability between the two sittings (Fig, 7) showed no significant difference in interpretation of the LM imaging (*p* = n.s. for both readers); however, a significant improvement in the reading of the other vessels probably suggests an early learning curve.

**Fig. 7:**
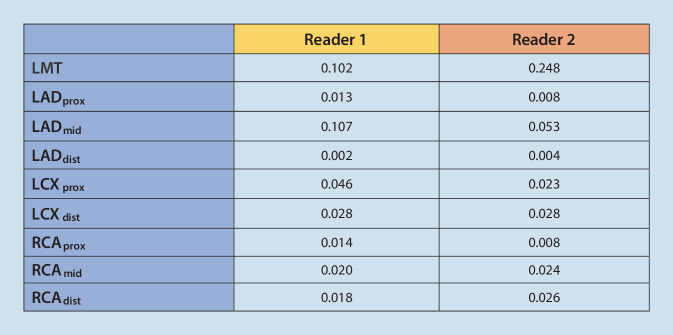
Intra-reader variability^a^ for the patient cohort. ^a^Wilcoxon test. *LMT* left main trunk, *LAD* left anterior descending, *LCX* left circumflex, *RCA* right coronary artery

## Discussion

The use of a CMRA 3D whole-heart sequence has been studied for its clinical applications in the diagnosis of CAD as it avoids the use of radiation and potentially nephrotoxic agents used in cCTA and ICA [8]. However, the process is hampered by long acquisition times and poor image quality. The results of our study show that CMRA with a sparse 3D whole-heart T1-weighted sequence with an acceleration factor of around 10, acquired in the waiting period between contrast administration and acquisition of the LGE sequences, renders good quality images with almost perfect inter-reader agreement in a reasonable amount of time. However, the image quality was shown to be poorer in the distal coronary segments as well as along the entire course of the LCX.

Early trials by Maintz et al. showed that coronary whole-heart sequences were only moderately inferior to cCTA in producing quality images for detecting CAD [3]. Another early study conducted by Sakuma et al., with 131 patients using the SSFP whole-heart CMRA approach, demonstrated moderate sensitivity and high specificity for the detection of significant narrowing in coronary segments of ≥ 2 mm in diameter [9]. A recent whole-heart CMRA study by Nakamura et al. showed the potential of this sequence in the noninvasive risk stratification of patients for major adverse cardiovascular events and cardiac death, while providing incremental prognostic value over conventional risk factors in those without history of myocardial infarction or prior coronary artery revascularization. The presence and severity of obstructive CAD detected by CMRA was associated with worse prognosis [10].

Studies have shown that high-resolution CMRA images can be reconstructed by exploiting the inherent 3D redundancies of the coronary artery anatomy and that a high degree of sparsity could be achieved by merging information. An early study by Yang et al. showed that the 3T CE whole-heart sequence could be completed in 9.0 ± 1.9 min, correctly identifying CAD in 32 out of 34 patients (sensitivity of 94.1%), while correctly ruling out CAD in 23 of 28 patients (specificity of 82.1%; [11]). Another study by Kato et al. showed that the acquisition of the whole-heart CMRA sequence could be completed with an average imaging time of 9.5 ± 3.5 min, and the ability to diagnose coronary stenoses was quite promising with a sensitivity of 88% and specificity of 72%. Data from the trial also revealed that CMRA could reliably rule out CAD with a negative predictive value (NPV) of 88% and left main disease with an NPV of 99% [12]. Similarly, Nagata et al. completed the whole-heart sequence in 6.2 ± 2.8 min with a reported sensitivity and specificity of 87% and 86%, respectively [13]. Our results showed that good-quality CMRA imaging with a sparse 3D whole-heart sequence, comparable to cCTA and/or ICA, was possible during the LGE waiting period and the procedure could be completed in 553 ± 46s.

The need for speed in the processing of a CMRA whole-heart sequence has meant that the quality of coronary images will be compromised. The results of our study reveal that although the proximal segments of the coronary tree could reproduce good quality images, which were comparable to images derived from cCTA and ICA, the distal end of these vessels and the LCX could not quite compare in quality. These results are similar to those reported in the study by Hamdan et al., wherein poorer image quality for the LCX was documented in the CMRA [6]. A possible explanation for this observation could be the relatively small caliber and posterior location of the LCX vessel. The increased distance between the artery and receiver coil results in a lower SNR, potentially contributing to this drawback.

The use of free-breathing techniques as used in our proposed framework has also been shown to be distinctly advantageous. For example, a free-breathing CMRA framework with a novel 3D-PROST reconstruction as proposed by Bustin et al. showed that sub-millimeter image acquisitions were possible in a fast and predictable scan time [14]. We performed the CE whole-heart sequence during the LGE waiting time in a pattern similar to a CMRA study conducted by Hirai et al., and were successful in rendering good quality images for the assessment of the coronary artery tree [15].

The potential application of this technique has interesting clinical implications. Current guidelines define the clinical management pathway for patients suspected of having CAD using a pre-test probability model [1]. Trials such as the International Study of Comparative Health Effectiveness with Medical and Invasive Approaches (ISCHEMIA) and Clinical Outcomes Utilizing Revascularization and Aggressive Drug Evaluation (COURAGE) have pertinently questioned the singular role of coronary anatomy in guiding clinical management of obstructive CAD. It is for this reason that the combination of noninvasive anatomical as well as functional diagnostic imaging for determining the relevance of coronary stenoses before any intervention has been proposed [16].

The use of myocardial perfusion imaging (MPI) in CMR was extensively studied in the CE-MARC trial and results showed that stress CMR could offer an accurate assessment of significant single-vessel and multivessel coronary disease [17]. A recent study by Zhang et al. revealed higher sensitivity, similar specificity, and higher accuracy on a per-patient basis for the combined use of CMRA and MPI compared to MPI alone [18]. These results establish the potential for differentiating between morphological and hemodynamically significant coronary stenoses within the framework of a single procedure.

Our results help us propose the integration of a whole-heart sequence during the LGE waiting time and especially during a stress CMR. The added anatomical information is of obvious benefit as it could help direct or avoid interventions in patients with angina resistant to optimal medical therapy.

### Study limitations

This single-center study is limited by the small number of patients, which influences the precision and statistical power of our analysis. This necessitates the confirmation of our results in a larger patient population. We did not measure vessel diameter or grade stenoses in the whole-heart CMRA sequence. The current whole-heart coronary MRA approach could be used for measuring luminal stenoses in the coronary arteries, as highlighted in other studies, but it would still not be able to provide sufficient information concerning atherosclerotic plaques in the arterial wall. Further studies are required to determine the accuracy of CMRA with radial k‑space sampling for measuring vessel diameter. The lack of data describing the patient cohort and their cardiovascular burden is also a significant limitation, which would need to be studied in further analyses.

## Conclusion

Coronary imaging with a sparse three-dimensional whole-heart sequence is feasible in a reasonable amount of time, producing good quality imaging. Image quality was poorer in distal coronary segments as well as along the entire course of the left circumflex artery.

## Supplementary Information

Supplementary Table. Patient demographics

## Declarations

For this article no studies with human participants or animals were performed by any of the authors. All studies performed were in accordance with the ethical standards indicated in each case.
