# Supplementary material for: Sparse 3D contrast-enhanced whole-heart imaging for coronary artery evaluation
Source: Herz. 2022 Jan 10;48(1):55–63. doi: 10.1007/s00059-021-05091-6 (PMC9892157; doi:10.1007/s00059-021-05091-6)
Supplement: Supplementary file 1 — Supplementary Table. Patient demographics [file 59_2021_5091_MOESM1_ESM.docx]

**Supplementary Table. Patient demographics**

| **Variable** | **Total population (n = 22)** |
| --- | --- |
| **Gender – Male, n (%) / female, n (%)** | 9 (40.9%) / 13 (59.1%) |
| **Age [years], median (IQR)** | 64 (24; 82) |
| **Body mass index [kg/m^2^],**  **median (IQR)** | 26.21 (19; 40) |
| **Coronary Artery Disease, n (%)**  **No coronary artery disease**  **Arteriosclerosis**  **Single-vessel disease**  **Triple-vessel disease** | 8 (36.3%)  3 (13.6%)  5 (22.7%)  6 (27.2%) |
| *IQR – interquartile range* | |
